# Dimethyl Fumarate‐Associated Acute Hepatocellular Injury Assessed for Causality Using the Updated RUCAM: A Biopsy‐Supported Case Report

**DOI:** 10.1002/ccr3.73430

**Published:** 2026-09-09

**Authors:** Ophir Lavon, Maram Salameh

**Affiliations:** ^1^ Rappaport Faculty of Medicine Technion‐Israel Institute of Technology Haifa Israel; ^2^ Clinical Pharmacology and Toxicology Unit Carmel Medical Center Haifa Israel; ^3^ Pharmacy Services Carmel Medical Center Haifa Israel

**Keywords:** case report, dimethyl fumarate, drug‐induced liver injury, hepatotoxicity, liver biopsy, multiple sclerosis, RUCAM

## Abstract

Dimethyl fumarate may rarely cause severe hepatocellular injury. In a 26‐year‐old woman, liver enzymes rose soon after treatment initiation, and biopsy supported DILI. Updated RUCAM assessment yielded 6, indicating probable causality, supported by dechallenge. Negative ANA reduced autoimmune hepatitis likelihood, although untested hepatitis E limited alternative‐cause assessment and causal certainty.

## 1 Introduction

Dimethyl fumarate (DMF) is an oral fumaric acid ester approved for relapsing forms of multiple sclerosis. The usual regimen begins with 120 mg twice daily for 7 days, followed by 240 mg twice daily [1]. In controlled trials, aminotransferase elevations were reported, most often without jaundice and with spontaneous resolution. Postmarketing experience subsequently identified clinically significant liver injury, and a liver injury warning is included in the prescribing information [1].

Published case reports and postmarketing analyses have described predominantly hepatocellular injury after DMF exposure [2, 3, 4, 5]. However, attribution is challenging in patients with multiple sclerosis because corticosteroids and other disease‐modifying treatments may be competing causes, and viral, autoimmune, ischemic, and biliary disorders must be considered. The updated Roussel Uclaf Causality Assessment Method (RUCAM) provides a structured, liver‐specific approach that scores latency, dechallenge, risk factors, concomitant drugs, exclusion of alternative causes, previous hepatotoxicity, and re‐exposure [6]. Current guidance also supports baseline measurement of aminotransferases, alkaline phosphatase, and bilirubin and repeat testing during therapy when clinically indicated [1, 7, 8].

We report a biopsy‐supported case of severe acute hepatocellular injury occurring shortly after DMF initiation and dose escalation and retrospectively assess the causal association using the 2016 updated RUCAM.

## 2 Case History/Examination

A 26‐year‐old woman with relapsing multiple sclerosis had normal liver biochemistry at the time of diagnosis. She initially received glatiramer acetate 20 mg subcutaneously once daily, but treatment was stopped after three injections because of a marked local reaction.

Several weeks later, she was hospitalized with an acute neurologic relapse manifested by diplopia and right‐sided paresthesia. She improved after intravenous methylprednisolone pulse therapy for 6 days, followed by an oral prednisone taper. Routine laboratory testing after corticosteroid therapy and before DMF initiation was unremarkable.

Two weeks later, DMF was started at 120 mg twice daily for 1 week and then increased to 240 mg twice daily. Ten days after dose escalation, routine biochemistry showed new aminotransferase elevations (ALT 674 U/L, AST 372 U/L, ALP 84 U/L). She was asymptomatic at the time of detection. DMF was discontinued immediately, and she was admitted shortly thereafter for evaluation. Physical examination on admission was unremarkable, without signs of hepatic encephalopathy or chronic liver disease.

**FIGURE 1:**
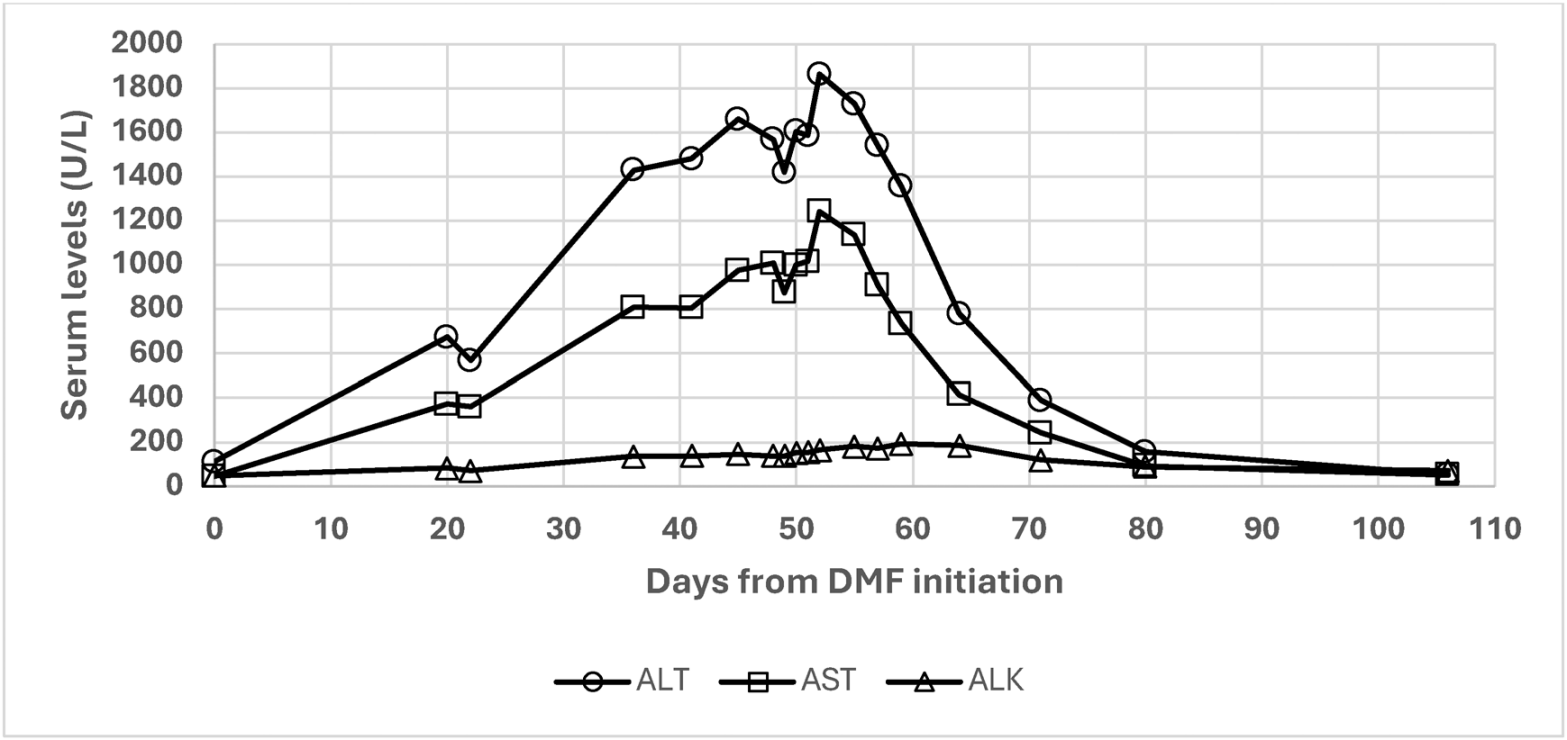
Liver enzymes serum levels. Serial liver biochemistry from dimethyl fumarate initiation through recovery. ALT peaked on day 45 and decreased by more than 50% of the peak excess above the upper limit of normal by day 57, supporting a + 2 updated RUCAM dechallenge score.

Admission testing showed a white blood cell count of 6000/μL, platelet count of 200 × 10^3^/μL, hemoglobin 13.7 g/dL, serum creatinine 0.65 mg/dL, and C‐reactive protein 0.12 mg/dL. Liver tests continued to worsen, reaching ALT 1569 U/L, AST 1009 U/L, ALP 139 U/L, and total bilirubin 1.94 mg/dL, while coagulation remained preserved (partial thromboplastin time 36.3 s; INR 1.23). Within a few days, she developed nausea, vomiting, anorexia, malaise, and fatigue. Peak liver biochemistry showed ALT 1864 U/L, AST 1242 U/L, and ALP 165 U/L, consistent with severe acute hepatocellular injury (Figure 1).

## 3 Differential Diagnosis, Investigations, Treatment, and Causality Assessment

The working diagnosis was acute hepatocellular DILI temporally associated with DMF. The principal competing diagnoses were acute viral hepatitis, autoimmune hepatitis, biliary obstruction, ischemic hepatitis, fatty liver disease with superimposed injury, glatiramer acetate‐associated injury, and corticosteroid‐associated DILI.

Serologic testing for hepatitis A, B, and C, Epstein–Barr virus, and cytomegalovirus was negative. Antinuclear antibody (ANA) testing was repeatedly negative, making autoimmune hepatitis less likely. Hepatitis E testing was not performed. Israeli seroepidemiologic data have suggested a low rate of ongoing hepatitis E virus transmission, particularly in younger persons, with no documented national outbreaks during the study period [9]. Nevertheless, hepatitis E could not be formally excluded for updated RUCAM scoring. Ultrasonography demonstrated a normal‐sized liver with fatty echotexture and no biliary obstruction. No hypotensive or ischemic event was documented, and the preserved INR and absence of encephalopathy argued against acute liver failure. Glatiramer acetate was considered less likely because exposure was brief and had ended several weeks before liver injury was detected. Methylprednisolone‐associated DILI is uncommon but has been reported in patients with multiple sclerosis [10]. Liver tests were unremarkable after corticosteroid therapy and before DMF initiation, but the prior corticosteroid exposure remained a clinically relevant alternative that could not be excluded with certainty.

**FIGURE 2:**
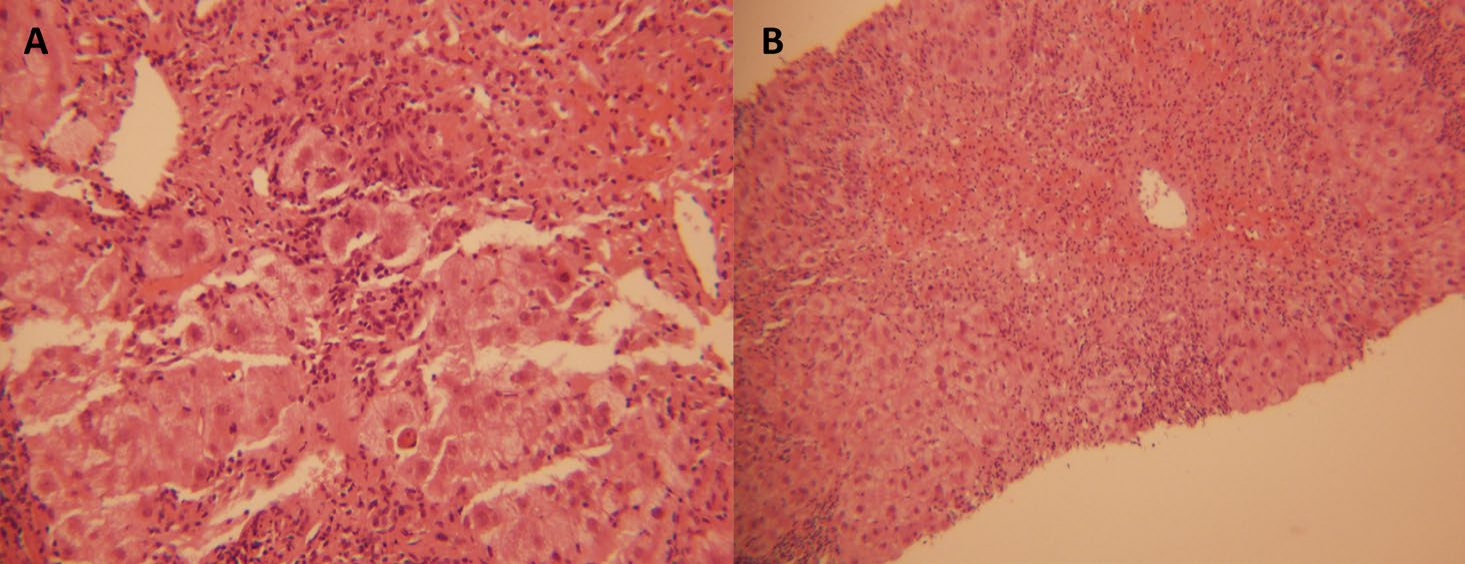
Liver biopsy. Representative liver biopsy micrographs (A: ×400; B: ×100) showing extensive perivenular necrosis, mixed inflammatory infiltrates, apoptotic bodies, and mild duct injury/proliferation supportive of acute drug‐induced liver injury.

Percutaneous liver biopsy was performed to characterize the injury pattern and assess alternative diagnoses. The biopsy showed disordered lobular architecture with extensive perivenular necrosis and fibrin deposition, predominantly mixed mononuclear inflammatory infiltrates, occasional apoptotic bodies, nuclear pleomorphism, occasional binucleation, rare mitotic figures, rare eosinophils in portal inflammation, reduced native bile ducts, and mild ductular proliferation (Figure 2). These findings were compatible with acute DILI in the appropriate clinical context, although histology cannot identify the responsible drug.

Treatment consisted of immediate discontinuation of DMF and supportive clinical and biochemical monitoring. No specific liver‐directed therapy was administered, and rechallenge with DMF was avoided.

Causality was retrospectively assessed using the 2016 updated RUCAM scale for hepatocellular injury [6]. The injury met the hepatocellular entry criterion because ALT exceeded 5 times the upper limit of normal while ALP remained within the reference range at initial detection. Only information documented in the available record and the complete serial liver‐enzyme trajectory shown in Figure 1 was scored. ALT reached its plotted peak on day 45 after treatment initiation and had decreased by more than 50% of the peak excess above the upper limit of normal by day 57, 12 days later. The dechallenge item therefore received +2 points. The alternative‐cause item remained 0 because only six of the seven Group I causes were formally ruled out, with hepatitis E untested. Repeatedly negative ANA reduced the likelihood of autoimmune hepatitis but did not change this item. The resulting total was 6, corresponding to probable causality (Table 1).

**TABLE 1:** 2016 updated RUCAM assessment.

Updated RUCAM item	Case‐specific assessment	Score
Time to onset from first DMF dose	Injury detected 17 days after initiation	+2
Course of ALT after cessation	ALT decreased by > 50% from the peak within 30 days (peak on day 45; > 50% decline documented by day 57)	+2
Risk factors	Age 26 years; no documented alcohol‐related risk factor	0
Concomitant drugs	Glatiramer acetate and corticosteroids discontinued > 15 days before detected onset; timing incompatible under updated RUCAM item	0
Search for alternative causes	HAV, HBV, HCV, EBV, and CMV tests negative; ultrasound excluded obstruction; no hypotensive event. ANA was repeatedly negative, reducing the likelihood of autoimmune hepatitis. HEV was not tested; therefore, only six of seven Group I causes were formally ruled out.	0
Previous hepatotoxicity of DMF	Clinically significant liver injury is included in product information	+2
Unintentional re‐exposure	No re‐exposure	0
Total	Probable causality	+6

*Note:* RUCAM interpretation: ≤ 0, excluded; 1–2, unlikely; 3–5, possible; 6–8, probable; ≥ 9, highly probable.

## 4 Conclusion and Results (Outcome and Follow‐Up)

After DMF withdrawal, the patient improved clinically and biochemically. The complete serial ALT trajectory in Figure 1 shows a peak on day 45 after treatment initiation, followed by a decrease of more than 50% of the peak excess above the upper limit of normal by day 57. This documented decline occurred within 30 days of the peak and fulfilled the updated RUCAM criterion for a + 2 dechallenge score. Liver enzyme values subsequently returned to the normal range within 10 weeks. She was discharged without hepatic sequelae, and no re‐exposure to DMF or methylprednisolone occurred during follow‐up.

The clinical course therefore represents severe acute hepatocellular injury in close temporal association with DMF therapy, with histologic support for acute DILI and complete recovery after withdrawal. The strength of attribution to DMF was evaluated separately using the updated RUCAM rather than inferred from the biopsy or dechallenge alone.

## 5 Discussion

The temporal relationship and biopsy findings support a diagnosis of acute hepatocellular DILI, but they do not establish DMF as the definitive culprit. Retrospective application of the 2016 updated RUCAM yielded a total score of 6, corresponding to probable causality (Table 1). The score was supported by a compatible 17‐day latency (+2), a greater than 50% ALT decrease from the peak within 30 days (+2) and labeled hepatotoxicity (+2). Repeatedly negative ANA reduced the likelihood of autoimmune hepatitis. However, hepatitis E was not tested. Although Israeli epidemiologic data suggest a low rate of ongoing hepatitis E transmission, especially in younger persons [9], epidemiologic probability does not replace formal virologic exclusion under the updated RUCAM. Accordingly, the alternative‐cause item remained 0, and the total score remained 6 (probable).

The histopathologic pattern remains an important feature of this report. Liver biopsy cannot prove that DMF was the culprit drug, but in selected cases it can support the type of injury and help evaluate competing diagnoses [7, 8]. In the present case, perivenular necrosis, lobular inflammation, apoptotic bodies, rare eosinophils, and mild duct injury and proliferation were compatible with acute DILI and aligned with the biochemical hepatocellular pattern.

Individual reports and an FDA‐led postmarketing analysis have described clinically significant liver injury after DMF initiation, with onset from the first days to several months and a predominantly hepatocellular pattern [2, 3, 4, 5]. However, the published cases were not uniformly assessed with the updated RUCAM, and differences in diagnostic completeness and causality methods limit direct comparison. The available literature therefore supports a pharmacovigilance signal and the product‐label warning but should not substitute for case‐specific exclusion of alternative causes.

Alternative explanations require critical consideration. Glatiramer acetate had been discontinued after only three injections and was temporally less compelling. The corticosteroid course had also ended before DMF initiation and therefore did not receive a negative concomitant‐drug score under the updated RUCAM timing criteria. Nevertheless, delayed methylprednisolone‐associated hepatotoxicity has been reported [10], so a contributory role cannot be excluded clinically. These uncertainties preclude definitive attribution, although the updated RUCAM score remains within the probable category.

The practical clinical message is therefore one of vigilance rather than definitive attribution. Clinicians should obtain baseline liver biochemistry, educate patients to report nausea, vomiting, anorexia, malaise, fatigue, jaundice, dark urine, or right upper quadrant pain, and promptly repeat liver tests when symptoms or abnormalities arise. When clinically significant DILI is suspected, interruption of DMF and systematic evaluation for alternative causes are appropriate [1, 7, 8].

The strengths of this report are the documented temporal sequence, the complete serial biochemical course, the greater than 50% ALT decline within 30 days of the peak, complete recovery after withdrawal, detailed histopathology, and transparent application of a structured causality method. The updated RUCAM table allows readers to identify which elements supported the probable association and which missing data constrained the assessment.

The principal limitations are the retrospective design and incomplete evaluation of alternative causes. Hepatitis E testing was unavailable. Consequently, the alternative‐cause item remained 0. Recent corticosteroid exposure also remained a clinically relevant competing explanation. Rechallenge was neither performed nor ethically indicated. RUCAM provides a structured probability category rather than proof of causation, and liver histology supports the diagnosis of acute DILI but cannot identify the responsible drug. The case should therefore be interpreted as biopsy‐supported acute hepatocellular DILI with a probable association with DMF by updated RUCAM (score 6), rather than as definitive proof of DMF‐induced injury.

## Author Contributions

**Maram Salameh:** data curation, investigation, writing – original draft, writing – review and editing. **Ophir Lavon:** conceptualization, writing – original draft, writing – review and editing, supervision, validation.

## Funding

The authors have nothing to report.

## Ethics Statement

The authors have nothing to report.

## Consent

Written informed consent from the patient was obtained according to journal guidelines.

## Conflicts of Interest

The authors declare no conflicts of interest.

## Data Availability

Data sharing not applicable to this article as no datasets were generated or analyzed during the current study.
